# Nurse practitioner led implementation of huddles for staff in long term care homes during the COVID-19 pandemic

**DOI:** 10.1186/s12877-023-04382-3

**Published:** 2023-11-02

**Authors:** Katherine S McGilton, Alexandra Krassikova, Aria Wills, Jennifer Bethell, Veronique Boscart, Astrid Escrig-Pinol, Andrea Iaboni, Shirin Vellani, Colleen Maxwell, Margaret Keatings, Steven C. Stewart, Souraya Sidani

**Affiliations:** 1 KITE Research Institute, Toronto Rehabilitation Institute, University Health Network, Toronto, Canada; 2 Lawrence S Bloomberg Faculty of Nursing, University of Toronto, Toronto, Canada; 3 School of Health and Life Sciences, Conestoga College, Kitchener, Canada; 4 Mar School of Nursing, Universitat Pompeu Fabra, Barcelona, Spain; 5 Social Determinants and Health Education Research Group, Hospital del Mar Medical Research Institute, Barcelona, Spain; 6 Department of Psychiatry, Temerty Faculty of Medicine, University of Toronto, Toronto, Canada; 7 Schools of Pharmacy and Public Health Sciences, University of Waterloo, Waterloo, Ontario Canada; 8 Faculty of Nursing, Toronto Metropolitan University, Toronto, Canada

**Keywords:** Long-term care, COVID-19, Moral distress, Healthcare providers, Nurse practitioner

## Abstract

**Background:**

Staff working in long-term care (LTC) homes during COVID-19 frequently reported a lack of communication, collaboration, and teamwork, all of which are associated with staff dissatisfaction, health concerns, lack of support and moral distress. Our study introduced regular huddles to support LTC staff during COVID-19, led by a Nurse Practitioner (NP). The objectives were to evaluate the process of huddle implementation and to examine differences in outcomes between categories of staff (direct care staff, allied care and support staff, and management) who attended huddles and those who did not.

**Methods:**

All staff and management at one LTC home (< 150 beds) in Ontario, Canada were included in this pre-experimental design study. The process evaluation used a huddle observation tool and focused on the dose (duration, frequency) and fidelity (NP’s adherence to the huddle guide) of implementation. The staff attending and non-attending huddles were compared on outcomes measured at post-test: job satisfaction, physical and mental health, perception of support received, and levels of moral distress. The outcomes were assessed with validated measures and compared between categories of staff using Bayesian models.

**Results:**

A total of 42 staff enrolled in the study (20 attending and 22 non-attending huddles). Forty-eight huddles were implemented by the NP over 15 weeks and lasted 15 min on average. Huddles were most commonly attended by direct care staff, followed by allied care/support, and management staff. All huddles adhered to the huddle guide as designed by the research team. Topics most often addressed during the huddles were related to resident care (46%) and staff well-being (34%). Differences were found between staff attending and non-attending huddles: direct care staff attending huddles reported lower levels of overall moral distress, and allied care and support staff attending huddles perceived higher levels of support from the NP.

**Conclusions:**

NP-led huddles in LTC homes may positively influence staff outcomes. The process evaluation provided some understanding of why the huddles may have been beneficial: the NP addressed resident care issues which were important to staff, encouraged a collaborative approach to solving issues on the unit, and discussed their well-being.

**Trial registration number:**

NCT05387213, registered on 24/05/2022.

**Supplementary Information:**

The online version contains supplementary material available at 10.1186/s12877-023-04382-3.

## Introduction

COVID-19 unleashed a series of catastrophic events in long-term care (LTC) homes, such as workforce shortages, inadequate resources, lack of communication and teamwork, and suboptimal infection prevention and control practices (IPAC); all these events have had negative consequences on LTC staff. Staff working in LTC homes throughout the pandemic cite a high prevalence of burnout [1], dissatisfaction with working conditions [2], post-traumatic stress, mood disturbances [3], lack of support [4] and moral distress related to not being able to provide the quality care in accordance with the residents’ wishes [5]. At the beginning of the COVID-19 pandemic, a team of international researchers provided considerations for LTC home leaders to support the health and well-being of LTC staff and residents, one of which was to incorporate huddles as a way to enhance timely team communication [6].

Huddles may involve a suite of interventions to support and facilitate changes in the work culture, by targeting topics that staff wish to discuss [7]. A recent scoping review of the effectiveness of huddles [8] indicated that in 67% of studies, huddles had a positive impact on team processes, including improvements in collaboration, communication across clinical roles and staff satisfaction. While the use of huddles is not widespread in LTC homes, they have been shown to positively influence resident and staff satisfaction [9]. Huddles can also create opportunities to provide timely advice to staff on current challenges in LTC homes, and to collaborate and discuss strategies to optimize resident care, which in turn may lead to improved teamwork and support [10]. In addition, huddles in LTC homes have been demonstrated to provide a forum where staff can discuss their struggles and generate strategies that address moral distress [11].

Challenges to the implementation of huddles in LTC homes include the identification of a facilitator, lack of standardized protocols and limited guidance for conducting the huddles [12]. The role of a competent facilitator should not be underestimated when implementing new practices, such as huddles, as the integration of practices in clinical settings can be very challenging [13]. The integrated Promoting Action on Research Implementation in Health Services (i-PARIHS) framework has been used to inform the implementation of best evidence into practice in prior intervention studies. The framework highlights facilitation as the active ingredient for implementing innovations into practice [14]. In this study, a nurse practitioner (NP) led the huddles based on evidence of NPs’ clinical and leadership facilitator roles in LTC homes highlighted during the pandemic [15]. NPs are advanced practice nurses with expertise to assess, diagnose and treat residents or patients [16], engage with family and care partners [17] and dedicate a large part of their time to coaching and educating staff [18]. As such, their scope of practice positions them well to build the capacity and expertise of staff in LTC homes [15].

An element essential for the success of huddles includes the process in which the huddles are implemented, the tasks achieved during the huddles and the discussions that are required for follow-up. Of the 24 studies included in a systematic review of multidisciplinary team huddles focused on patient safety [19], only two included the evaluation of the huddles [20, 21]. These studies reported on the fidelity of huddle implementation, which was quantified in terms of participants’ attendance and/or completion of a checklist on the topics covered or activities performed during the huddle. Evaluating the implementation process of huddles is essential to generate knowledge that helps understand how, when, and why interventions succeed or fail [22].

Conducting huddles also requires careful consideration of the intended target audience to ensure the right staff complements are engaged in the process. In their study, Wagner et al. [10] provided evidence that often, direct care staff were not active participants in care planning meetings or shift reports and suggested that huddles can provide a unique opportunity for direct care staff to discuss challenges and generate solutions. In this study, huddles were designed to engage direct care, allied care and support, and management staff, as each of these categories can contribute meaningfully to the resolution of challenges. The involvement of direct care staff and allied care and support staff in resident care provides opportunities to identify relevant practice-related issues. Management staff’s participation is important when administrative challenges (e.g., staffing or equipment shortages) arise or to demonstrate their support of the huddles. We expected differential influences of the huddles on outcomes for these three different staff categories in anticipation of their variable attendance due to sick leaves, outbreaks and frequent changes in policies and directives. Variability in exposure to the intervention’s dose has been found to affect the level of outcome achievement [22].

The specific objectives of this study were to (1) evaluate the process of huddles implementation; (2) compare the staff who attended the huddles and the staff who did not attend the huddles on outcomes of moral distress, job satisfaction, physical and mental health, and perceptions of support provided by the NP; and (3) explore potential differentials impacts of the intervention on outcomes reported by the three staff categories.

## Methods

### Design

We used a pre-experimental static-group comparison design to determine differences in outcomes between healthcare providers who participated in the huddles and those who did not [23], stratified by the staff category.

The intervention group included staff who attended any number of huddles, further referred to as the ‘attendees’, whereas the control group included staff who did not attend any huddles, or ‘non-attendees’. While we had originally set out to conduct a quasi-experimental design, we encountered significant issues related to staff turnover, absenteeism, and stress, which limited the ability to collect pre- and post-outcome data from the same individuals. Ethics approval for the project was obtained from the Toronto Rehabilitation Institute, University Health Network Ethics Board, REB#20-6298. All methods were carried out per relevant guidelines and regulations.

### Setting and sample

The huddles took place on two units in a privately owned, not-for-profit LTC home with < 150 beds, located in Ontario, Canada. The LTC home consisted of five units in total, with 29 residents residing on each unit. All units provided long-term care services, including dementia care. The NP was employed on a contract basis, and whose role included providing care to all residents, support to families, management, and staff. The NP was at the home 8 h a week in addition to providing acute, episodic care as part of an acute care outreach team. The target population consisted of three staff categories. The category of direct care staff was personal support workers (PSWs), registered practical nurses (RPNs), and registered nurses (RNs). Allied health care professionals included physiotherapists (PTs), occupational therapists (OTs), registered dietitians, and rehabilitation/physiotherapy assistants. Support staff encompassed recreation and life enrichment staff, environmental services, behavioural support staff, and resident support aids (a role introduced throughout the pandemic to assist PSWs in non-care related tasks). Management staff included administrators, nurse managers, human resources, and administrative assistants. Allied healthcare professionals and support staff were combined in the analysis because of the small number of respondents within each type. All staff were included if they worked any shift on a casual, part-time, or full-time basis.

### Intervention

Forty-eight huddles were implemented between May and August 2021. The NP facilitated huddles twice a week for staff on the day (1:45–2:00 pm) and evening (2:00–2:15 pm) shifts. A flyer with the huddle information and times was posted at the nursing station. Prior to the implementation of the intervention, the NP received training in facilitating the huddles. The NP was presented with a toolkit containing resources to effectively carry out huddles, an explanation of the huddle structure and scripts for delivering the structured huddle, and documentation and reflection sheets [7].

In this study, huddles differed from shift reports as they focused on involving staff from all disciplines to collectively address opportunities for improvement identified by staff, be that clinical care, housekeeping, dietary etc. with the aim of ensuring a timely response. Specifically, ‘opportunity for improvement cards’ were made available for staff to complete anonymously throughout the implementation window to identify their needs; the completed cards were posted on a whiteboard to be addressed at the next huddles. Occasionally, based on previous huddle discussions, additional facilitators with expertise on the topic were invited to co-lead the huddle with support from the NP for example.

### Data collection

#### Survey recruitment

After the 15-week huddle period, all staff working at the LTC home were invited to complete a survey via email sent by the LTC home management through the home’s newsletter. When participants accessed the link for the online survey, they were prompted to read the consent information and the survey instructions and to confirm their willingness to participate in the research study. Once consented, they were able to access the survey questions, which included their sociodemographic characteristics, whether they participated in the huddles (yes or no), and outcome data. Staff who completed the survey had the option to be included in a draw for one of five $50 e-gift cards.

#### Process evaluation

The process evaluation included assessments of the key aspects of dose and fidelity with which the huddles were implemented: (1) duration of the huddles (in minutes); (2) attendance (number of participants and their staff category); (3) frequency of delivery (dates of huddle occurrence); and (4) adherence, by the NP facilitator, to the structure of the huddles (huddle topic and aim, environment, collaborative culture, and risk management plan, i.e., the plan going forward to address any concerns that arose). The dose and fidelity of the intervention implementation were assessed using the Huddle Observation Tool (HOT); a structured tool originally developed by Edbrooke-Childs et al. [7] for paediatrics, which was adapted to the context of LTC by the study team. The NP leading the huddle completed the HOT and collected detailed notes reflecting on the huddle, in particular on collaborative culture and risk management plans developed during the huddle (Appendix A).

#### Outcome measures

All outcome data were collected via an online survey distributed within 6 weeks of the final huddle. Three reminder emails to complete the survey were sent. Self-reported data were collected on:

1. Moral distress, using a ten-item checklist adapted from the Moral Distress in Dementia Care Instrument [24]. The checklist was adapted by Iaboni et al., and pilot tested with staff working in LTC homes during COVID-19 to measure the negative feelings experienced when they are unable to do what they believe is the right thing for a resident [5]. The 10 items inquire about causes or situations leading to staff moral distress. Respondents were asked to rate the distress experienced on a 5-point scale ranging from none at all (1) to an extremely large amount of distress (5). As done by previous researchers [5], the responses to each item were analyzed separately due to the independence of the items’ content, that is, staff may encounter different causes or situations leading to distress.
2. Support provided by the NP who facilitated the huddles, using 5 items Supportive Supervisory Scale [25] on a 5-point scale, from never (1) to always (5). A total score was computed as the sum of the 5 items, where higher scores indicate the NP as more supportive. The scale was previously validated with RN supervisors in LTC [25]. Given that NPs are graduate-prepared RNs with leadership responsibilities, the attributes evaluated by the scale are in line with what is required of NPs, specifically being reliable and empathic.
3. Job satisfaction, using a single item asking “how satisfied are you overall with your current job in the LTC home” rated on a 4-point scale, where higher scores indicate more satisfaction. This measure has high reliability and validity [26] and has been used previously in studies involving LTC homes [27].
4. Overall health (In general, how would you say your health is?) and mental health (In general, how would you say your mental health is?) status of survey respondents were measured with the above two items from Statistics Canada [28], using a 5-point scale, ranging from poor (1) to excellent (5).

### Data analysis

Data on staff’s demographic characteristics and the huddle dose and fidelity were summarized using descriptive statistics. Cross-tabulations and Fisher’s exact test were used to examine differences in the characteristics of attendees and non-attendees. Bayesian inference was chosen for accurate multiple comparisons [29] and is useful even when the sample size is small [30]. For each outcome, a Bayesian proportional odds model was used to estimate the posterior probability distribution of the difference in responses for attendees and non-attendees [31]. For each situation contributing to moral distress, the proportion of the posterior probability distribution less than zero was used to estimate the probability of a low level of moral distress. For the other outcomes, the proportion of the posterior probability distribution greater than zero was used to estimate the probability of a high level of support, satisfaction, or health. For this interval hypothesis testing, a probability of 0.95 or higher was considered statistical evidence. Bayesian analyses were started from uninformative priors and stratified by category of staff (i.e., direct care, allied care/support, or management). Stata 16 was used for statistical calculation.

To determine the topics discussed in the huddles, an exploratory quantitative content analysis was selected [32]. The content analysis of the detailed notes documented by the NP in the risk management planning section of the HOT tool was carried out by two members of the research team. The two coders (AK and AW) independently reviewed the data line-by-line assigning codes to capture the topics discussed, followed by a meeting to discuss, and reconcile discrepancies.

## Results

### Sample

A total of 42 individuals completed the online survey post-intervention. Of these, 33% were direct care staff, 48% were allied care and support staff, and 19% were management staff. Overall, most respondents were between the ages of 18 and 34 (n = 15, 36%), self-identified as white (n = 38, 90%), women (n = 35, 83%), and worked more than four days a week (n = 26, 62%). Nearly half of the survey respondents had been working between 1 and 5 years at the LTC home (n = 21, 50%).

Twenty participants indicated that they attended the huddles; these participants included two management staff, six direct care staff and 12 allied care and support staff. Twenty-two respondents indicated not participating in the huddles, of which six were management staff, eight direct care staff, and eight allied care and support staff. There were no statistically significant between-group differences in the staff characteristics (Table 1).

**Table 1:** Characteristics of staff completing surveys

Characteristic	Frequency (column %)			*P*-value
	Overall (n = 42)	Huddles (n = 20)	No huddles (n = 22)	
**Age (years)**				0.275
18–34	15 (36%)	9 (45%)	6 (27%)	
35–44	9 (21%)	5 (25%)	4 (18%)	
45–55+	18 (43%)	6 (30%)	12 (55%)	
**Gender**				0.444
Women	35 (83%)	16 (80%)	19 (86%)	
Men	7 (17%)	4 (20%)	3 (14%)	
**Race**				0.144
White	38 (90%)	19 (95%)	19 (86%)	
Non-white	3 (7%)	0	3 (14%)	
Missing	1 (2%)	1 (5%)	0	
**Role** ^*^				0.264
Direct care	14 (33%)	6 (30%)	8 (36%)	
Allied care/support	20 (48%)	12 (60%)	8 (36%)	
Management	8 (19%)	2 (10%)	6 (27%)	
**Experience in current role in the facility (years)**				0.343
< 1	8 (19%)	3 (15%)	5 (23%)	
1–5	21 (50%)	12 (60%)	9 (41%)	
6–15	6 (14%)	4 (20%)	3 (14%)	
16–20 +	6 (14%)	1 (5%)	5 (23%)	
**Work schedule**				0.118
< 2 days	4 (10%)	0	4 (18%)	
2–4 days	12 (29%)	5 (25%)	7 (32%)	
> 4 days	26 (62%)	15 (75%)	11 (50%)	
**Redeployment**	17 (41%)	10 (50%)	7 (32%)	0.188
**Overall moral distress**				0.254
An extremely large amount	3 (7%)	3 (15%)	0	
A large amount	14 (33%)	6 (30%)	8 (36%)	
A moderate amount	14 (33%)	5 (25%)	9 (41%)	
A small amount or none at all	11 (27%)	6 (30%)	5 (23%)	
**Overall NP support**				0.842
Always	12 (29%)	6 (30%)	6 (29%)	
Often	12 (29%)	7 (35%)	5 (24%)	
Occasionally	13 (32%)	5 (25%)	8 (38%)	
Seldom or never	4 (10%)	2 (10%)	2 (9%)	
**Work satisfaction**				0.769
Strongly satisfied	3 (7%)	2 (10%)	1 (5%)	
Satisfied	31 (74%)	15 (75%)	16 (73%)	
Dissatisfied or strongly dissatisfied	8 (19%)	3 (15%)	5 (22%)	
**Physical Health**				0.041
Excellent	11 (26%)	8 (40%)	3 (14%)	
Very good	7 (17%)	1 (5%)	6 (27%)	
Good	16 (38%)	6 (30%)	10 (46%)	
Fair	5 (12%)	2 (10%)	3 (14%)	
Poor	3 (7%)	3 (15%)	0	
**Mental Health**				0.228
Excellent	3 (7%)	3 (15%)	0	
Very good	8 (19%)	4 (20%)	4 (18%)	
Good	13 (31%)	4 (20%)	9 (41%)	
Fair	14 (33%)	6 (30%)	8 (36%)	
Poor	4 (10%)	3 (15%)	1 (5%)	

### Process evaluation of the intervention

A total of 48 huddles were held from May to August 2021. The mean duration of the huddles was 15 min, with a 10–30-minute range and huddle attendance ranged between two to 13 attendees per huddle. The dose of the intervention to which the three categories of staff were exposed differed slightly: direct care staff attended all huddles, whereas management and administrative staff were present at only 23% of the huddles. For the first three weeks of implementation, the huddles occurred between three to four times a week but were later reduced to twice per week based on feedback from the staff. Eleven of the huddles were co-led by other staff (e.g., housekeeping, rehabilitation lead, recreation, and life enrichment) with support from the NP. In terms of fidelity, more than 90% of the huddles followed the pre-defined structure. See further details in Table 2.

As identified through the content analysis, topics most frequently discussed were related to resident care (n = 22, 46%). Perceived positive improvements to resident care were documented by the NP. For example, when a resident was in isolation and experienced a fall and psychological symptoms of dementia, direct care staff shared and discussed successful approaches to engage the resident and minimize their responsive behaviours and risk of another fall. In another huddle, through collaborative work between direct care staff, dietary aids, and a dietitian, changes were made to a resident’s meal plan which led to the resident enjoying meals again. Other examples of resident care-related discussions included the NP providing staff with education to optimize person-centred care by sharing strategies to support residents who were anxious about receiving care, and education to better understand residents’ diagnoses and medications. As per the notes provided by the NP, solutions developed in the huddles likely led to improvements in residents’ quality of life and the work environment of the staff.

The second most common topic addressed at the huddles was related to staff well-being (n = 16, 34%). Discussions focused on the risks of neglecting self-care, addressing staff members’ feelings of anxiety associated with providing resident care, discussing the prevalence of post-traumatic stress as well as validating frustration and concerns related to the pandemic to boost staff morale. Some huddles provided an opportunity for celebration such as lifting restrictions on family visits in the home. Additional topics covered in the huddles were related to IPAC practices and measures (n = 4, 8%), including the emphasis on the importance of following IPAC guidelines; the use of proper equipment (n = 2, 4%); and security (n = 2, 4%), which consisted of a review of safety plans associated with an emergency at the home.

**Table 2:** Characteristics of huddles led by the nurse practitioner at the LTC home

	**Huddles** (n = 48)	
**Duration (mins) mean, range**	15 (10–30)	
**Shift**		
Day	28 (58%)	
Evening	20 (42%)	
**Facilitator**		
Nurse practitioner	40 (83%)	
Other^*^	8 (17%)	
**Attendance**	8 (2–13)	
Direct care	48 (100%), range 1–7	
PSW	47 (98%), range 2–5	
RPN	46 (96%), range 0–2	
RN	3 (6%), range 0–1	
Allied care/support	34 (71%), range 1–7	
Management	11 (23%), range 0–1	
**Huddle Structure Adherence**		
Aim stated	46 (96%)	
Clear leader	45 (94%)	
Positive event shared	44 (92%)	
Looking back	45 (94%)	
Looking now	44 (92%)	
Planning	43 (90%)	

Note. PSW, Personal Support Worker; RPN, Registered Practical Nurse; RN, registered nurse

^*^ Other facilitators that co-led the huddles with the NP included housekeeping, rehabilitation lead, recreation and life-enrichment staff

### Staff outcomes

Survey responses indicated that 40% of the staff were experiencing a large or extremely large level of moral distress. The following situations were primarily associated with a large amount of moral distress: restrictions on family visits, seeing care levels decrease due to staffing shortages and high staff turnover. Of note, 50% of attendees reported experiencing a high level of moral distress associated with a lack of support for their own safety when providing care to residents exhibiting challenging behaviours related to dementia, compared to 32% of non-attendees.

Differences emerged when comparing levels of moral distress for the three categories of attendees and non-attendees. Direct care attendees reported a lower level of overall moral distress as compared to non-attendees (posterior probability = 0.9933). In addition, attendees reported low levels of moral distress associated with seeing lower-quality resident care due to high staff turnover (posterior probability = 0.9909) and having to follow COVID-19-related policies that don’t seem to benefit residents (posterior probability = 0.9780) compared to non-attendees. Management staff attending huddles reported higher levels of moral distress associated with high staff turnover (posterior probability = 0.001), not having enough activities for residents (posterior probability = 0.03), and not having enough staff to provide care (posterior probability = 0.0003), as compared to non-attendees. See further details in Table 3.

**Table 3:** Proportion of the posterior probability distribution consistent with less moral distress, overall and stratified by role

Type of moral distress reported post-implementation	Huddles vs. no huddles			
	**Overall** (n = 42)	**Direct care** (n = 14)	**Allied care/support** (n = 20)	**Management** (n = 8)
Overall, how much, if any, moral distress do you currently feel in your job	0.71	.**99**	0.19	0.09
Seeing the care suffer for residents with or without dementia because of high staff turnover or new staff without the training to provide dementia care	0.32	.**99**	0.06	.*001*
Having to follow COVID-19 related policies or procedures even when they don’t seem best for the residents	0.87	.**98**	0.51	0.21
Seeing a low quality of life for residents with or without dementia because there are not enough activities	0.12	0.94	.*01*	.*03*
Seeing residents with or without dementia suffering from pain or other symptoms because they are not treated appropriately	0.58	0.92	0.47	0.06
Seeing the care suffer for residents with or without dementia because of the effects of restrictions on family visits	0.45	0.81	0.29	0.10
Seeing poor care for a resident with or without dementia because of poor communication between staff members	0.32	0.62	0.53	0.12
Not reporting what I believe is neglect or abuse of a resident with or without dementia because I feel no one listens or I’m afraid of causing trouble	0.40	0.60	0.78	0
Having to provide care to aggressive residents with or without dementia without the supports I need to feel safe	0.21	0.53	0.34	0
Seeing the care suffer for residents with or without dementia because physicians do not visit often enough	0.57	0.47	0.86	0.21
Seeing the care suffer for residents with or without dementia because there are not enough staff to do the work	0.10	0.37	0.21	.*0003*
Total score	0.36	0.84	0.32	.*03*

Note: Rows are ordered by the estimated probability less moral distress was reported by respondents in Direct care who participated in huddles compared to those in Direct care who did not participate in huddles

In bold is probability of 0.95 or higher, which is considered statistical evidence for lessened moral distress in those attending the huddles

In italics is probability of 0.05 or lower which is considered statistical evidence of more moral distress in those attending the huddles

In terms of perceived support from the NP, 58% of all participants reported experiencing support from the NP often or always. The majority of attendees believed the NP struck a balance between their and residents’ concerns, compared to 45% of non-attendees. For direct care staff, there were no differences in perceptions of support between the attendees and non-attendees, whereas allied care and support staff in attendance reported greater support from the NP (posterior probability = 0.9642). Management staff attendees reported higher levels of support from the NP related to the NP being open to remarks (posterior probability = 0.9998) and striking a balance between resident concerns and those of the managers (posterior probability 0.9998). In terms of job satisfaction, as well as physical and mental health, there was no evidence of differences between the attendees and non-attendees, for direct care staff and allied care and support staff. However, managers who attended huddles reported worse physical health (posterior probability = 0.04) and mental health (posterior probability = 0.003) (Table 4).

**Table 4:** Proportion of the posterior probability distribution consistent with greater support from the nurse practitioner, work satisfaction, health and mental health, overall and stratified by role

Type of Nurse Practitioner support	Huddles vs. no huddles			
	Overall (n = 42)	Direct care (n = 14)	Allied care/support (n = 20)	Management (n = 7)
My Nurse Practitioner strikes a balance between resident/families’ concerns and mine	0.89	0.31	.**972**	.**9979**
I can rely on my Nurse Practitioner to be open to any remarks I may make to him/her	0.79	0.53	0.76	.**9998**
‍I can rely on my Nurse Practitioner when I ask for help, for example, if things are not going well between myself and residents and/or their families	0.77	0.80	0.77	0.57
My Nurse Practitioner keeps me informed of any decisions that were made in regard to my residents	0.41	0.26	0.81	0.49
My Nurse Practitioner keeps me informed of any major changes in the work environment of organization	0.33	0.19	0.69	0.73
Total score	0.69	0.32	.**964**	0.70
**Work satisfaction and health**				
Work satisfaction	0.81	0.84	0.53	0.34
Health	0.59	0.52	0.77	*0.04*
Mental Health	0.63	0.64	0.71	*0.003*

Note: Rows are ordered by evidence of strengthened Nurse Practitioner support

In bold is probability of 0.95 or higher, which is considered strong statistical evidence for strengthened support

In italics is probability of 0.05 or lower which is considered statistical evidence of more moral distress in those attending the huddles

## Discussion

The aim of this study was to evaluate the process of implementing the huddles and examine whether there were differences in outcomes of moral distress, job satisfaction, physical and mental health, and the perceived amount of support provided by the NP between huddles attendees and non-attendees. Huddles were facilitated by the NP over 15 weeks, with 2 huddles per week on average, including both day and evening staff. In line with previous research [10], we found that huddles were most often targeted at concerns related to resident care. Additionally in this study, the NP facilitator allowed staff to discuss topics focused on their well-being and self-care strategies given the COVID-19 pandemic, which staff appreciated. Differences in outcomes were found for direct care staff attending the huddles, as they reported lower levels of overall moral distress compared to direct care staff who did not attend the huddles. Allied care/support staff attending the huddles perceived the NP as more supportive, compared to non-attendees.

The huddles were targeted at direct care staff and as such were attended most often by PSWs and RPNs, who represent the majority of LTC staff responsible for resident care [33] but who may be limited in opportunities to share their perspective [10]. Allied care and support staff were also invited to attend, as communication amongst disciplines is key to trust and promoting a positive, person-centred culture in the LTC home [34]. The input from all team members greatly enhanced the amount and type of solutions offered concerning resident care. Evidence suggests that solutions developed in huddles have been associated with improved resident outcomes [35], and a reduction in responsive incidents [10]. Ensuring LTC homes utilized the expertise of all team members has been demonstrated to be essential for the excellent quality of care, and an interdisciplinary approach to resident care should be considered in the design of future huddles in this setting [36–38].

Moral challenges have arisen specifically due to the COVID-19 pandemic, as staff have reported distress regarding ruptured family connections, resident loneliness and the increasingly ambiguous and rapidly changing expectations of staff roles and responsibilities [39]. These concerns were corroborated by our findings. Staff have identified the need to vent frustrations and discuss their moral distress openly to process and learn from their experiences [40]. Imposing a structured process and a safe medium can facilitate honest and open-reflections more effectively than informal discussions among coworkers [40]. Similarly in our study, huddles provided an opportunity for staff to discuss their fatigue, concerns about resident care and COVID-19, and in some instances make changes to improve resident care and their situations. Our findings provide preliminary evidence that staff who attended the huddles compared to those who did not, reported a lower amount of moral distress, specifically related to seeing residents’ care levels decrease because of staffing issues, lack of training, and COVID-19 policies. However, caution is warranted in interpreting these findings given that it is possible that those staff with lower moral distress self-selected to attend the huddles. As the pandemic has left LTC homes increasingly vulnerable, huddles are one solution to continue supporting staff as the LTC sector recovers.

NPs are uniquely positioned, because of their role, to facilitate the huddles. NPs have an important clinical and leadership facilitator role in LTC homes that was clearly highlighted during the pandemic [15]. Facilitators who help implement best practices are required in LTC homes, as similar to most healthcare settings, this sector is frequently under-resourced and often lacks leaders with the experience and skills required to introduce and implement best practices. Engaging a dedicated facilitator, like an NP, to enable the facilitation of evidence-based innovations into practice may be essential for implementation [14]. Results from our study supported the important facilitator role the NP took on to lead the huddles and their influence on positive outcomes, as allied care and support staff who attended the huddles perceived NP’s support as higher than those who did not attend the huddles. Being able to attend the huddles allowed allied and support staff more opportunities to discuss resident care situations with the NP, which was most likely welcomed as there are usually few opportunities to have collaborative problem-solving opportunities with the NP and direct care staff outside of the huddles. Given the majority of LTC homes do not have an NP, leaders within the home need to be identified who can facilitate these huddles, including, but not limited to, managers, charge nurses, and personal support workers.

Results on fidelity, specifically attendance records, indicate that management staff had a limited presence at the huddles, which could be related to there being few managers in the building. In an effort to engage the managers, they were included as part of the planning committee and their involvement in the huddles was anticipated. However, due to the realities of the COVID-19 pandemic and staff shortages, managers had competing priorities and were unable to regularly attend the huddles. The attendance of managers at huddles is important, as many issues causing moral distress were rooted in staff concerns associated with staffing shortages and COVID-19 fatigue and having a manager present may have facilitated organizational responses. Furthermore, in this study managers attending the huddles reported more moral distress and health concerns than those not attending, which may have negatively impacted their ability to hear staff’s concerns further affecting their distress levels. Some LTC home managers have been described as having passive-avoidant leadership styles [41], possibly due to the overwhelming administrative work within the context of limited resources, without any capacity for more negativity from staff. Whereas strong leadership among managers in LTC homes, characterized as closely monitoring work and coaching and giving feedback, has been shown to lessen job strain and improve social support among direct care staff [42]. Future research should focus on training and experience requirements for developing LTC home leaders, understanding the management staff’s role within the huddles; as well, as strategies to communicate staff’s concerns and suggested solutions to the management team if they are unable to attend.

### Strengths and limitations

The major strength of this study was the implementation of a structured huddle intervention which was adapted based on previous research and delivered with high fidelity. Additionally, the use of a knowledgeable facilitator, such as the NP, to implement the huddles was a strength. While the study provides important insights, it is not without limitations. We do not know how frequently each staff member attended the huddles, as this information was not collected due to anonymity concerns, and therefore we could not evaluate the true dose of the intervention for each huddle attendee. Although we know the number of huddle attendees, we are unable to determine the response rate compared to the total number of staff working, as we cannot account for unfilled shifts due to staff shortages experienced by the LTC home. In this LTC home setting, huddles were only held during day and evening shifts, limiting the opportunity for staff who worked primarily during the night shift to participate. However, staff who were not able to attend the huddles were able to track and contribute to the issues and solutions discussed using the whiteboards. The design of the study was adjusted to decrease the burden on staff working at the LTC home. Due to continuous COVID-19 outbreaks and staff turnover, staff were unable to complete the surveys as expected, so we were unable to evaluate and compare changes in staff outcomes over time or know if attendees and not attendees differed regarding their characteristics; making the findings of this study difficult to generalize to other LTC homes. In addition, staff who participated in the study were predominately white, which is not representative of staffing in most LTC homes. Finally, multiple outcomes were assessed with small sample size, however, statistical procedures were used to address these issues. Future studies should examine changes in outcomes between staff participating in huddles.

## Conclusion

The process evaluation provided evidence that the NP followed the structured protocol for conducting a huddle. Huddles provided a forum for staff to discuss issues which were meaningful to them, including patient care concerns and issues related to their well-being. There was some evidence that those who attended the huddles experienced less moral distress and greater support from the NP. NPs are important members of LTC home teams and can be instrumental in implementing evidence-based practices. Additional studies employing robust methodological approaches are needed to demonstrate how huddles can impact resident outcomes and staff retention.

### Electronic supplementary material

Below is the link to the electronic supplementary material.

Supplementary Material 1

## Data Availability

The datasets generated and analyzed in this study are available from the corresponding author on reasonable request.

## List of abbreviations

HOT: huddle observation tool
IPAC: infection prevention and control
LTC: Long-Term Care
NP: nurse practitioner
OT: occupational therapist
PSW: personal support worker
PT: physiotherapist
RN: registered nurse
RPN: registered practical nurse
