# Supplementary material for: Nurse practitioner led implementation of huddles for staff in long term care homes during the COVID-19 pandemic
Source: BMC Geriatr. 2023 Nov 2;23:713. doi: 10.1186/s12877-023-04382-3 (PMC10623826; doi:10.1186/s12877-023-04382-3)
Supplement: Supplementary file 1 — Supplementary Material 1 [file 12877_2023_4382_MOESM1_ESM.docx]

Appendix 1. HOT tool [7] adapted to the context of the LTC home.

| Leader  Initials: | | | |  | Event name:  (e.g.,. ‘SBAR Communication Framework’) | | | |
| --- | --- | --- | --- | --- | --- | --- | --- | --- |
|  | | | |  |  | | | |
| Unit  Bed occupancy |  | | |  | Number of attendees by role:  In-house / Agency | | | Completed  (please tick) |
| Date | / / | | |  | NP |  |  |  |
| Start time: | : AM / PM | | |  | RN |  |  |  |
| End time: | : | | |  | RPN |  |  |  |
|  | | | |  | PSW |  |  |  |
| Were the following discussed? | | Yes | No |  | Other: |  |  |  |
| Aim of the huddle | |  |  |  |  |  |  |  |
| A positive event | |  |  |  |  |  |  |  |
| Looking back | |  |  |  |  |  |  |  |
| Looking now | |  |  |  |  |  |  |  |
| Planning | |  |  |  |  |  |  |  |
|  | |  |  |  |  |  |  |  |
| *If you would like to add to or expand on these categories, please use the additional notes sections.* | | | |  |  |  |  |  |
| During today’s huddle | |  |  |  |  |  |  |  |
| Was a clear leader identified? | |  |  |  |  |  |  |  |
|  | |  |  |  |  |  |  |  |

| **To what extend would you disagree or agree with the following statements about today’s huddle?**  Please tick one statement for each row | | | | | |
| --- | --- | --- | --- | --- | --- |
|  | Strongly disagree | Disagree | Neither agree nor disagree | Agree | Strongly agree |
| **Structure**  The huddle followed a clear structure |  |  |  |  |  |
| **Environment**  The Huddle was disrupted by external distractions or interruptions |  |  |  |  |  |
| **Collaborative culture**  Everyone had the opportunity to contribute, and all points of view were respected |  |  |  |  |  |
| **Risk Management**  There were opportunities to identify risks and discuss concrete plans to mitigate the risks |  |  |  |  |  |

| **Notes on observations and reasons for rating given** | |
| --- | --- |
| **Structure:** | **Environment:** |
| **Collaborative culture:** | **Risk management:** |
| **Additional notes:** | |
